# Melanoma Cancer Therapy Using PEGylated Nanoparticles and Semiconductor Laser

**DOI:** 10.34172/apb.2022.055

**Published:** 2021-07-03

**Authors:** Abdorreza Asrar, Zahra Sobhani, Mohammad Ali Behnam

**Affiliations:** ^1^Faculty of Naval Aviation, Malek Ashtar University of Technology, Iran.; ^2^Quality Control Department, Faculty of Pharmacy, Shiraz University of Medical Sciences, Shiraz, Iran.; ^3^Nano Opto-Electronic Research Center, Electrical and Electronics Engineering Department, Shiraz University of Technology, Shiraz, Iran.

**Keywords:** Photothermal therapy, Carbon nanotube, TiO2 NPs, Melanoma, Hyperthermia, Cancer therapy

## Abstract

***Purpose:*** Photothermal therapy (PTT) is a procedure that converts laser beam energy to heat so can disturb tumor cells. Carbon nanotubes (CNTs) have unique properties in absorption optical energy and could change optical power into heat in PTT procedures. Additionally, titanium dioxide (TiO_2_) nanoparticles (NPs) have a unique feature in absorbing and scattering light. Therefore, these mentioned NPs could play a synergistic role in the PTT method.

***Methods:*** CNTs and TiO_2_ NPs were injected into the melanoma tumor sites of cancerous mice. Then sites were excited using the laser beam (λ = 808 nm, P = 2 W, and I = 4 W/cm^2^). Injected NPs caused hyperthermia in solid tumors. Tumor size assay, statistical analysis, and histopathological study of the treated cases were performed to assess the role of mentioned NPs in PTT of murine melanoma cancer.

***Results:*** The results showed that CNTs performed better than TiO_2_ NPs in destroying murine melanoma cancer cells in animals.

***Conclusion:*** The present study compared the photothermal activity of excited CNTs and TiO_2_ NPs in cancer therapy at the near-infrared spectrum of light. Tumors were destroyed selectively because of their weakened heat resistance versus normal tissue. PTT of malignant melanoma through CNTs caused remarkable necrosis into the tumor tissues versus TiO_2_ NPs.

## Introduction

Nanoparticles (NPs) have broad applications in photonics, chemical sensing, drug delivery, and imaging. Carbon nanotubes (CNTs) and plasmonic NPs have unique properties in absorbing and scattering light. They tuned to absorb laser beam energy at a specific range of wavelengths.^1^

Titanium dioxide (TiO_2_) appears in the world as familiar mineral crystals such as anatase and rutile.^2^ TiO_2_ is efficient to transform solar energy into electrical energy.^3^ TiO_2_ NPs have substantial effects on the near-infrared (NIR), visible, and UV regions of the photonic spectrum. These specific properties qualify them to use in solar cells,^4^ optic biosensors, cancer therapy,^5,6^ biological and medical applications. TiO_2_ is a biocompatible material to use in cosmetic products such as sunscreens. This metal oxide has antibacterial, anti-fungal, and anti-cancer activity because of its potency in the production of free radicals through excitation with different light spectra. These unique properties have led to select TiO_2_ NPs as suitable candidates for photothermal therapy (PTT).

Recently, a generation of an innovative category of photothermal NPs has introduced a new cancer therapy method called PTT.^7,8^ PTT is a superior procedure among other ways to treat cancer.^9^ Semiconductor laser based on NIR beam could penetrate the skin very quickly and causes controlled temperature increment in a target zone to ablate tumors and cancerous cells. This phenomenon is called hyperthermia.^10^ NPs are considerably useful as agents to generate heat in tumor sites because of their high absorption cross-sections and high photostability.^11^ In addition to the heat efficiency of these NPs, their biocompatibility, and low toxicity are of great importance. Sometimes cytotoxicity of the injected NPs may cause death.^12^ Laser excitation of NPs, localized in the tumor sites, could increase tumor tissues’ temperature and generate heat to eradicate tumors by causing protein denaturation and membrane lysis, and make necrosis and apoptosis in the tumor sites.^13^ NIR light (800 nm-1300 nm) could enter the body and barely is attenuated by biological systems.^3,10^

CNTs have several characteristics, including good optical absorption, good electrical conductivity, high thermal conductivity, and strong mechanical strength, unique for nanotechnology and bio-engineering.^10,14^ CNTs have several applications in drug delivery systems and could act as a carrier for drugs, imaging agents, and antigens.^15,16^ The optical absorbance spectrum of CNTs indicates exceptional plasmonic characteristics dependent on the structure of CNTs.^16,17^ Also, some studies have represented that CNTs could use in both drug delivery systems and PTT technique.^1,10^

Functionalization of NPs with a biocompatible and hydrophilic polymer such as polyethylene glycol (PEG) has been utilized to increase dispersibility and cell entrance properties of the NPs.^18^

In the present study, the photothermal activity of NIR-excited CNTs and TiO_2_ NPs in destroying melanoma cancer was compared. After tumor inoculation in mice, injection of mentioned NPs in tumors, and laser excitation of tumor sites, the tumor size was measured and the efficacy of mentioned NPs in destroying tumor cells was assessed.

## Materials and Methods

### Preparation of CNT-PEG_4000_

Multi-walled CNTs (number of walls: 10-25, outer diameter: 10-25 nm, length: 2000-4000 nm) and PEG_4000_ were purchased respectively from Plasmachem (Germany) and Sigma-Aldrich (USA).

Sixty-four milligrams of CNTs was added to 32 mL of deionized water. Then 640 mg PEG_4000_^19^ was added to the mixture. The dispersion was sonicated for about 20 minutes and was stirred overnight at 25°C. After stirring, the suspension was centrifuged at 4200 rpm for 8 minutes, and the supernatant was collected for further analysis.^1,10^ The formation of the PEG layer around the CNTs confirmed using field emission scanning electron microscopic method (FESEM; Czech Republic).

### Preparation of TiO_2_-PEG_4000_ NPs

TiO_2_ NPs (with 15-20 nm diameter) were purchased from the US Research Nanomaterials Company (USA). Their purity was more than 99%, with the anatase phase. 64 mg of TiO_2_ NPs was added to 32 mL of deionized water. Then 640 mg PEG_4000_^20^ was added to the mixture. The dispersion was sonicated for about 20 minutes and was stirred overnight at 25°C. After stirring, the suspension was centrifuged at 4200 rpm for 8 minutes, and the supernatant was collected to be further analyzed.^1,10^ The formation of the PEG_4000_ layer around TiO_2_ NPs was confirmed using a transmission electron microscope (TEM) (Philips Electron Optics, the Netherlands).

Cytotoxicity Assay of NPs

The cytotoxicity of CNT-PEG_4000_ and TiO_2_-PEG_4000_ was evaluated by standard dimethylthiazole-tetrazolium (MTT) assay. Human hepatocellular carcinoma cell line ((HepG2), Pasteur Institute, Iran) was cultured in RPMI-1640 medium (Shellmax, China), then supplemented with 1.5% penicillin-streptomycin (Invitrogen, USA) and 15% fetal bovine serum (Shellmax, China) at 37°C in a humidified incubator with 10% CO_2_. Cells in the exponential growth phase were seeded in 96-well plates at the density of 1×10^4^ viable cells/well. They encountered different concentrations of TiO_2_-PEG_4000_ and CNT-PEG_4000_. After 24 hours of incubation, 20μl of MTT (5 mg/mL) and 100 μL of the medium was added. The prepared plates were incubated for 6 hours. The formazan crystals were dissolved in 100 µL of dimethyl sulfoxide. Then, plates were read at 575 nm against 695 nm on an ELISA reader. Cell viability was evaluated considering a decrement of values from a dimethyl sulfoxide control.

### Animals and housing

A metastatic murine melanoma (B16/F10) cell line was ordered from the National Cell Bank of Iran Pasteur Institute. B16/F10 cell lines were cultured in RPMI 1640 medium, under 7% CO_2_ at 37°C. After that, the cultured cell line was fed with 10% fetal bovine serum, 100 IU/mL of penicillin, and 100µg/mL streptomycin.^21^ Inbred C57 female mice weighing 15-30 g with ages of 5-6 weeks were purchased from the Animal Laboratory of Shiraz University of Medical Sciences, Shiraz, Iran. They were randomly allocated into four balanced groups (N = 5). Melanoma cells at the number of 4.5×10^5^ were suspended in 180 µL culture medium and injected into the right flank of inbred mice, subcutaneously.^22^

The methods, protocols, and procedures of this research were accomplished according to the guidelines of the Animal Care Committee of the Iran Veterinary Organization. The experiments were performed under the same techniques for all mice. This research was proved by the Ethical Committee of Shiraz University of Medical Sciences.

The mice were anesthetized by a combination of ketamine and xylazine. Tumor sizes were measured before the start of the treatment and four days after that utilizing an ultrasonography machine (Ultrasonix SonixOP; Canada) and a caliper. The tumor size was computed using the below equation:

Tumor volume = (L/2) * W^2^ (mm^3^)^23^ L: length, W: Width

Mice were treated according to the below instruction:

In group Ι (CNT), 0.15 mL CNT-PEG_4000_ (2 mg/mL) was injected into the mice intratumorally.

In group ΙΙ (TiO_2_ NPs), 0.15 mL TiO_2_ NPs-PEG_4000_ (2 mg/mL) was injected into the mice intratumorally.

Group ΙΙΙ (Laser Therapy), did not get any NPs.

Group ΙV (Control), did not get any pretreatment.

Groups Ι, ΙΙ and ΙΙΙ were faced with a NIR continuous-wave laser diode^24,25^ (wavelength of 808 nm, power of 2 W, and intensity of 4 W/cm^2^)^26^ for 9 minutes in two days after the beginning of the treatment.

## Histopathological evaluation

Four days after the beginning of the treatment, all cases were sacrificed. Their excised masses were sent for histopathologic examination.^10^ The formalin-fixed paraffin-embedded blocks were provided, and slides were marked with hematoxylin and eosin (H&E) procedure.^1,3^ The samples were checked for microscopic scrutiny.

## Statistical analysis

**The data were exhibited as mean ± standard deviation. The significant difference was statistically evaluated by paired-sample** *t* test. Comparisons at different times were evaluated by ANOVA with repeated measures. Statistical analyses were done by SPSS® version 20. The *P* value < 0.05 was considered indicative.

## Results and Discussion

**Figure 1:**
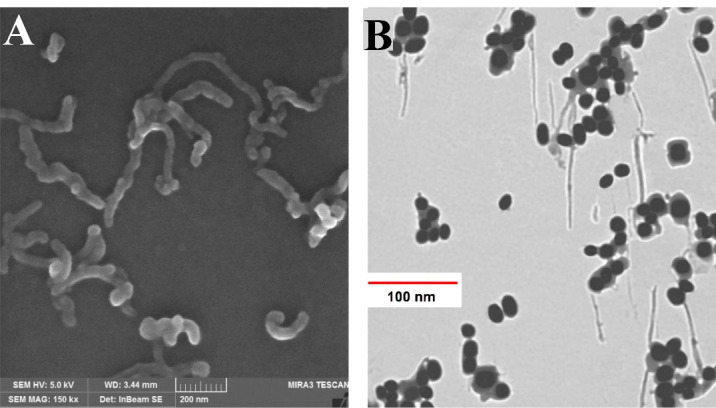
Electron microscopy images of different NPs. (**A**) FESEM image of CNT-PEG_4000_ and (**B**) TEM image of TiO_2_-PEG_4000_ NPs.

Morphological pictures of CNT-PEG_4000_ and TiO_2_-PEG_4000_ are shown in FESEM and TEM images (Figure 1A, B). According to this figure, a thin layer surrounds the CNTs and TiO_2_ NPs, which confirms the presence of PEG on the surface of mentioned NPs. This figure shows a continuous layer of biocompatible and hydrophilic polymer (PEG_4000_) with an approximate thickness of 12nm around the surface of CNTs and TiO_2_ NPs. Coating of NPs with PEG_4000_ not only increases their aqueous dispersibility and biocompatibility but also decreases their toxicity. PEGylated NPs for delivery of anti-cancer drugs have better activity against tumor cells due to enhanced permeability and retention effect after intravenous injection.^27^

**Figure 2:**
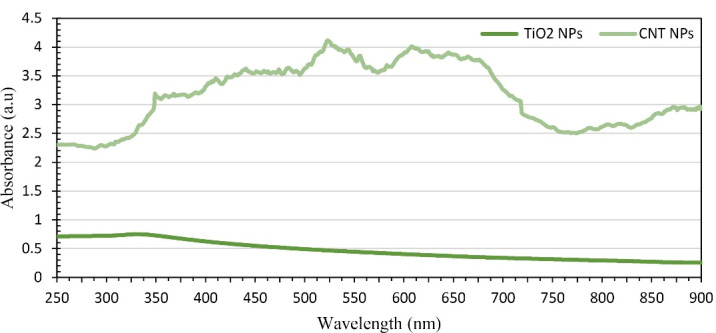
UV-Vis light absorption spectrum of CNT-PEG_4000_ and TiO_2_-PEG_4000_ NPs.

Figure 2 represents the UV-Visible absorption spectrums of CNT-PEG_4000_ and TiO_2_-PEG_4000_ NPs. The highest absorption wavelength of CNT-PEG_4000_ is in the range of NIR and visible spectra. However, TiO_2_-PEG_4000_ NPs have lower optical absorption than CNT-PEG_4000_. This considerable difference interprets the higher plasmonic properties of CNTs in the NIR and visible spectra.

## Cytotoxicity of PEGylated CNTs and TiO_2_ NPs

**Figure 3:**
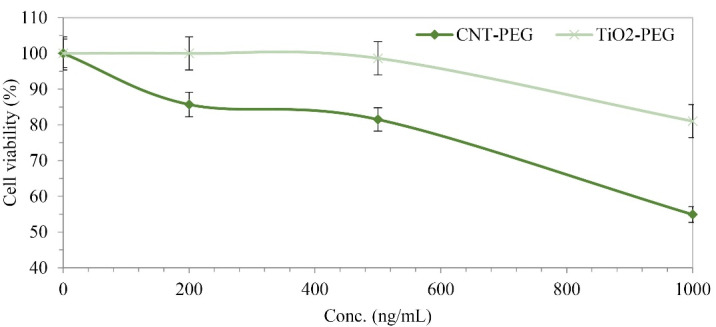
MTT assay of CNT-PEG_4000_ and TiO_2_-PEG_4000_ against HepG2 human cell line (*P* < 0.05).

**The cytotoxicity outline of CNT-PEG**_4000_ and TiO_2_-PEG_4000_ against cultured cell lines were assessed. HepG2 human cell line was exposed to the mentioned NPs at various concentrations for 24 hours. Figure 3 demonstrates that at concentrations up to 1000 ng/mL, the cytotoxicity of CNT-PEG_4000_ NPs was higher than TiO_2_-PEG_4000_. According to our investigations, the presence of PEG on the surface of CNTs and TiO_2_ NPs improved the cell viability of mentioned NPs significantly.^10,20^ Coating of CNTs and TiO_2_ NPs with PEG extended the cell viability in the HepG2 cell line.

## Photothermal therapy of tumors

**Figure 4:**
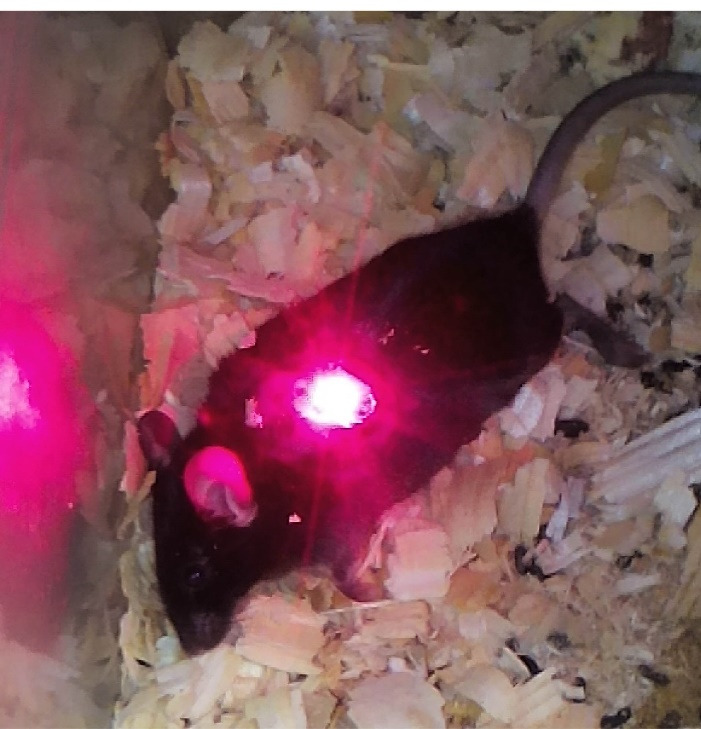
Irradiation of melanoma tumor in mouse.

**B16/F10 murine melanoma cell line was injected subcutaneously to the female C57BL/6J inbred mice to initiate tumor growth. After two weeks, tumors were grown sufficiently to start treatment. After grouping and injection of CNT-PEG**_4000_ and TiO_2_-PEG_4000_, the *in vivo* effects of mentioned NPs combined with laser excitation on the tumor size were checked. According to Figure 4, laser excitation of tumors is accomplished to cover all around the tumor sites by optimizing the spot size of the laser diode.

**Figure 5:**
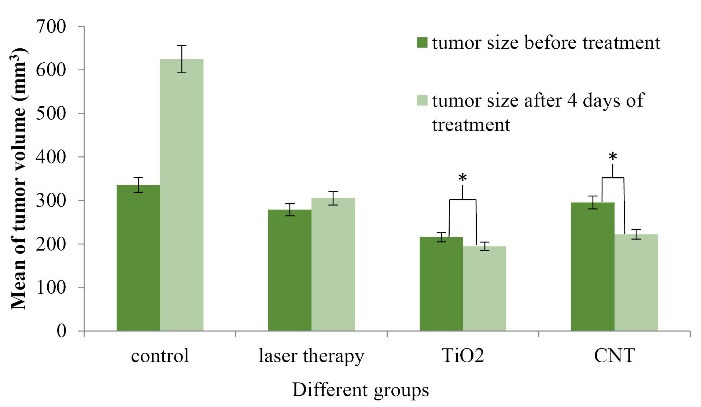
Tumor sizes in various groups (before and four days after the beginning of treatment with PTT procedure) (N = 5 in every group, * indicates *P* < 0.05).

The tumor sizes were measured before and 4 days after the treatment (Figure 5). Collected data were analyzed and showed a significant difference between groups Ι and ΙΙ. The tumor sizes in CNT and TiO_2_ groups were shrunk noticeably. The tumor growth was faster in the Control group than the Laser Therapy group; however, in CNT and TiO_2_ groups, the tumor sizes were reduced remarkably. By injecting CNT-PEG_4000_ and TiO_2_-PEG_4000_ NPs to the tumor sites and laser excitation of them, the tumor sizes were decreased in CNT and TiO_2_ groups (*P* < 0.05).

This process indicates that the average tumor size before and four days after the beginning of the treatment is extended in the Control group, but these sizes are shrunk in CNT and TiO_2_ groups. *P* value is significantly different between all groups. The gradient of tumor size reduction in CNT and TiO_2_ groups is discussable. Ultrasound images were taken for all cases located in different groups to determine the volume of tumors.

**Figure 6:**
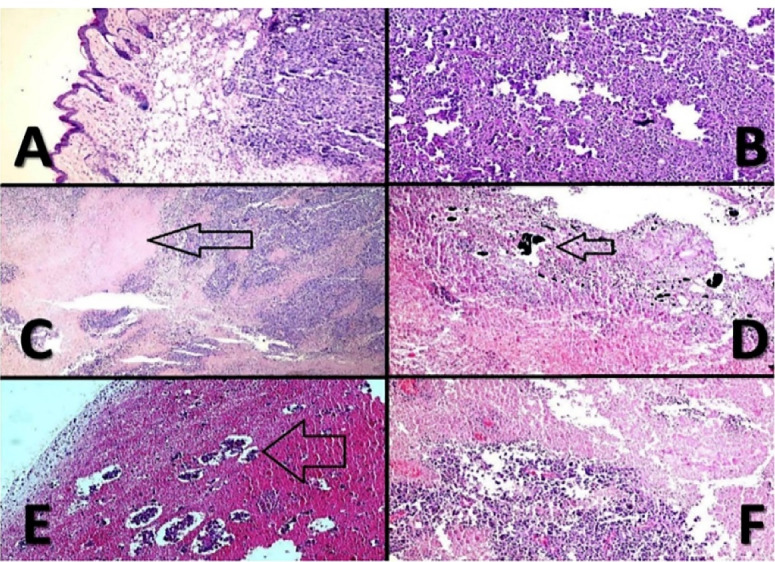
Malignant melanoma. (**A**); histopathologic evaluation of different cases indicates that the tumor centered in upper dermis (X100, H & E). (**B**); histomorphology of the cases shows sheets of tumor cells with minimal melanin pigments (X100, H & E). (**C**); geographic necrosis (arrow) in CNT-PEG_4000_ cases (X100, H & E). (**D**); NPs in necrotic areas, (arrow) (X100, H & E). (**E**); extensive necrosis with islands of tumor cells in TiO_2_-PEG_4000_ cases; (arrow) (X100, H & E). (**F**); severe necrosis in CNT-PEG_4000_ cases (X100, H & E).

For professional scrutiny, a histopathologic examination was carried out. Gross assessment of cases clarified severe decrement of tumor sizes in CNT and TiO_2_ groups versus Laser Therapy and Control groups. The microscopic review indicated the appearance of nodular subtype malignant murine melanoma in whole specimens. Necrosis was the best indicator among CNT and TiO_2_ cases, and its percentage was more significant in CNT samples versus TiO_2_ cases. As shown in Figure 6, there was a direct relationship between the percentage of necrosis and the presence of CNT and TiO_2_ NPs. In Control cases, mitosis was very high, and totally, no indication of ulceration, regressive fibrosis, vascular invasion, lymphocytic infiltration, neurotropism, and microsatellites were found in treated samples. Table 1 represents the results in detail.

**Table 1:** Results of histopathologic examination

**Groups**	**Necrosis (%)**	**Mitotic rate**
CNT	85%	< 1/mm^2^
TiO_2_	45%	< 1/mm^2^
Laser Therapy	25%	< 1/mm^2^
Control	5%	> 1/mm^2^

When NPs were injected into the tumor sites and irradiated by a semiconductor laser, the electrons located in the NPs forced to transport from the ground states to the excited states. Therefore, absorbed photon energy changed into heat through a various photophysical process that includes electron-photon and electron-electron relaxation.^28,29^ Results assessment of this study indicated that, after irradiation of NPs through a semiconductor laser, CNT-PEG_4000_ caused tumor necrosis more efficiently than TiO_2_-PEG_4000_ NPs in the murine melanoma cancer model. This result can be discussed by the UV-Vis absorption diagram of mentioned NPs. CNT-PEG_4000_ has higher plasmonic properties than TiO_2_-PEG_4000_ NPs in the visible-NIR range.

Laser excitation elevated the local temperature through increase hot electrons temperature in NPs. CNT-PEG_4000_ NPs generated heat more efficiently than TiO_2_-PEG_4000_ NPs. The results of *in vivo* studies demonstrated that in CNT and TiO_2_ groups, the tumor growth was stopped and the tumor size was shrunk, while in the control group the tumor growth was continuous. Furthermore, histopathological assessment represented 85% and 45% necrosis in CNT and TiO_2_ groups, respectively, which indicates the high capability of CNT-PEG_4000_ versus TiO_2_-PEG_4000_ NPs as agents in the PTT procedure.

The results demonstrated that PEGylation of mentioned NPs could enhance their biocompatibility and hydrophilicity in the body and, consequently, enhance the tumor penetration of NPs in the PTT procedure. CNTs exhibit advantages and limitations in PTT when compared to TiO_2_ NPs. In the PTT procedure, the efficacy of CNTs in treating local melanoma tumors was better than TiO_2_ NPs, while the cytotoxicity of TiO_2_ NPs was lower than CNTs.^10,24^ Thermal activity of CNTs against cancer models in mice and rabbits was reviewed. Still, potency evaluation of TiO_2_ NPs in this field is limited to studies on the diverse cell lines, such as cervical cancer cells, bladder cancer cells, adenocarcinoma cells, monocytic leukemia cells, colon carcinoma cells, breast epithelial cancer cells, and human hepatoma cells.^1,3,27,30,31^

## Conclusion

The local elevating temperature in malignant tissues is an efficient technique in cancer therapy. Exposure to high temperature for a sufficient time causes protein denaturation and membrane lysis and can increase oxidative stress. These effects lead to coagulative necrosis or apoptosis. Herein, CNT-PEG_4000_ and TiO_2_-PEG_4000_ NPs have been assessed to define their effects in PTT. Laser excitation of CNT-PEG_4000_ versus TiO_2_-PEG_4000_ NPs represents the better performance of CNT-PEG_4000_ via PTT procedure in extirpating murine melanoma cancer model. Functionalization of CNTs and TiO_2_ NPs could improve their efficiency in both PTT and drug delivery system because mentioned NPs, supply a versatile platform to coincidentally deliver heat and drugs with facile control to the cancer cells.

## Acknowledgments

This study was supported by Shiraz University of Medical Sciences, Shiraz, Iran as a research project (approval number 12079). We would like to appreciate Dr. Fatemeh Behnam, Doctor of Veterinary Medicine at Shiraz University for her helpful comments.

## Ethical Issues

The manuscript has not been published previously. It is not divided into different parts, and no data have been fabricated or manipulated. All authors agree to submit this manuscript to this journal. Authors have contributed sufficiently to this experimental work, and they are responsible for the results.

International, national, and institutional guidelines for the care and use of animals were followed in this study. This experiment was performed in the Center of Experimental and Comparative Medicine, Shiraz University of Medical Sciences, under the relevant regulatory standards. It was also approved by the Ethical Committee of Shiraz University of Medical Sciences.

## Conflict of Interest

The authors declare that they have no conflict of interest.
